# Genetic Diversity of *Plasmodium falciparum* Populations in Malaria Declining Areas of Sabah, East Malaysia

**DOI:** 10.1371/journal.pone.0152415

**Published:** 2016-03-29

**Authors:** Mohd Ridzuan Mohd Abd Razak, Umi Rubiah Sastu, Nor Azrina Norahmad, Abass Abdul-Karim, Amirrudin Muhammad, Prem Kumar Muniandy, Jenarun Jelip, Christina Rundi, Mallika Imwong, Rose Nani Mudin, Noor Rain Abdullah

**Affiliations:** 1 Herbal Medicine Research Center, Institute for Medical Research, Kuala Lumpur, Malaysia; 2 Sabah State Health Department, Rumah Persekutuan, Kota Kinabalu, Sabah, Malaysia; 3 Zonal Public Health Laboratory, Tamale Teaching Hospital, Tamale, Northern Region, Ghana, West Africa; 4 Department of Molecular Tropical Medicine and Genetics, Faculty of Tropical Medicine, Mahidol University, Bangkok, Thailand; 5 Vector Borne Disease Sector, Disease Control Division, Ministry of Health, Federal Government Administrative Centre, Putrajaya, Malaysia; Université Pierre et Marie Curie, FRANCE

## Abstract

Malaysia has a national goal to eliminate malaria by 2020. Understanding the genetic diversity of malaria parasites in residual transmission foci can provide invaluable information which may inform the intervention strategies used to reach elimination targets. This study was conducted to determine the genetic diversity level of *P*. *falciparum* isolates in malaria residual foci areas of Sabah. Malaria active case detection was conducted in Kalabakan and Kota Marudu. All individuals in the study sites were screened for malaria infection by rapid diagnostic test. Blood from *P*. *falciparum*-infected individuals were collected on filter paper prior to DNA extraction. Genotyping was performed using merozoite surface protein-1 (MSP-1), merozoite surface protein-2 (MSP-2), glutamate rich protein (GLURP) and 10 neutral microsatellite loci markers. The size of alleles, multiplicity of infection (MOI), mean number of alleles (*Na*), expected heterozygosity (*He*), linkage disequilibrium (LD) and genetic differentiation (F_ST_) were determined. In Kalabakan, the MSP-1 and MSP-2 alleles were predominantly K1 and FC27 family types, respectively. The GLURP genotype VI (751–800 bp) was predominant. The MOI for MSP-1 and MSP-2 were 1.65 and 1.20, respectively. The *Na* per microsatellite locus was 1.70. The *He* values for MSP-1, MSP-2, GLURP and neutral microsatellites were 0.17, 0.37, 0.70 and 0.33, respectively. In Kota Marudu, the MSP-1 and MSP-2 alleles were predominantly MAD20 and 3D7 family types, respectively. The GLURP genotype IV (651–700 bp) was predominant. The MOI for both MSP-1 and MSP-2 was 1.05. The *Na* per microsatellite locus was 3.60. The *He* values for MSP-1, MSP-2, GLURP and neutral microsatellites were 0.24, 0.25, 0.69 and 0.30, respectively. A significant LD was observed in Kalabakan (0.495, *p*<0.01) and Kota Marudu *P*. *falciparum* populations (0.601, *p*<0.01). High genetic differentiation between Kalabakan and Kota Marudu *P*. *falciparum* populations was observed (F_ST_ = 0.532). The genetic data from the present study highlighted the limited diversity and contrasting genetic pattern of *P*. *falciparum* populations in the malaria declining areas of Sabah.

## Introduction

Globally, 3.3 billion people are at risk of malaria. In 2015, the number of new malaria cases reached 214 million and malaria deaths were estimated to reach 438,000 [1]. Between 2000 and 2015, malaria incidence and mortality rates decreased worldwide by 37% and 60%, respectively. Scaling up interventions such as the widespread usage of insecticide-treated bed nets (ITN), indoor residual spraying (IRS), larval control, improved diagnostic testing and treatment by artemisinin combination therapy (ACT) have contributed to the decrease in malaria cases worldwide. An increasing number of countries including Malaysia are in the process of eliminating malaria [1].

After implementing the World Health Organization’s Global Malaria Eradication Program (1967–1981) and Malaria Control Program (1982–2010), Malaysia has managed to substantially decrease the malaria cases and enter the pre-elimination phase, with the Malaysian government now aiming to eliminate malaria by the year 2020. During the implementation of the National Malaria Elimination Plan (2011-current), indigenous human malaria cases were further decreased to only 606 cases in 2014 (Vector Borne Disease Control Sector, Ministry of Health, 2014). Sabah, East Malaysia, contributed 93% (561 cases) of indigenous human malaria cases in 2014 (Vector Borne Disease Control Sector, Ministry of Health, Malaysia, 2014). Up until November 2015, indigenous human malaria cases in Sabah have dropped to 207 cases as compared to 522 cases reported in November 2014 (Vector Borne Disease Control Sector, Ministry of Health, Malaysia, 2015). On the lead up to malaria elimination, understanding the genetic diversity and population structure of the residual malaria parasite populations is crucial to guide monitoring and evaluation of malaria control strategies and antimalarial interventions.

The *P*. *falciparum* genetic diversity is usually performed through genotyping of polymorphic region of antigenic markers such as block 2 of the merozoite surface protein 1 (MSP-1), block 3 of the merozoite surface protein 2 (MSP-2) and RII repeat region of the glutamate rich protein (GLURP) [2]. Another genotyping approach is the multilocus genotyping that targets non-antigenic markers such as single nucleotide polymorphisms (SNPs) [3, 4] and simple sequence repeats of neutral microsatellites [5, 6]. Using these molecular genotyping approaches, the factors contributing to the *P*. *falciparum* population structure and diversity such as inbreeding, epidemic population expansion, geographic isolation and gene flow or introgression of foreign parasites can be characterized. Several genetic measurements such as expected heterozygosity (*He*), the proportion of polymorphic loci, the number of alleles (*Na*), multiplicity of infection (MOI), linkage disequilibrium (LD) and F-statistics (F^ST^) are used to estimate the factors affecting the malaria parasite population structure [2, 5–8]. In addition, the genetic diversity and population structure studies can be used to monitor the effects of any malaria scale-up interventions, such as the impact of malaria control and elimination programs [9].

Effective malaria control measures have successfully reduced malaria transmission in many hyperendemic regions of sub-Saharan Africa. In future, the population structure in these regions are expected to become similar to the low transmission regions like Southeast Asia and South America. The declining malaria transmission, as a result of scaling up interventions, has been shown to affect the genetic diversity pattern and population structure of *P*. *falciparum* [3, 9–11]. The genetic diversity of *P*. *falciparum* has previously been studied in a variety of transmission setting regions such as Africa, South America and Southeast Asia [5, 11–15]. High malaria transmission settings like those found in African regions normally report *P*. *falciparum* populations with high level of *He*, high MOI value, low or insignificant linkage disequilibrium (LD) and low genetic differentiation (F_ST_) [5, 16]. In contrast, low or limited genetic diversity pattern such as low *He* and MOI, high or significant LD and high F_ST_ are mostly reported in the regions with low transmission setting such as Southeast Asia and South America [5, 11–13, 17].

The effect of malaria control interventions on the *P*. *falciparum* population structure in Malaysia could not be assessed due to the lack of genetic data and systematic genetic surveillance study. A recent genotyping based on MSP-1 and MSP-2 allelic families has demonstrated low levels of diversity in *P*. *falciparum* populations in Pahang, Peninsular Malaysia (West Malaysia) which reflected the low transmission level in the area [17]. Multilocus genotyping based on neutral microsatellites has also revealed the contrasting genetic structure of *P*. *falciparum* populations in several parts of Sabah indicating that they are genetically different from each other [11]. Herein, we report genetic diversity data for *P*. *falciparum* isolates collected during the malaria active case detection studies conducted in the remaining residual foci areas of Sabah, Kalabakan and Kota Marudu, by genotyping the highly polymorphic parasite antigenic markers (MSP-1, MSP-2 and GLURP) and 10 neutral microsatellite markers.

## Materials and Methods

### Study Area and Population

**Fig 1:**
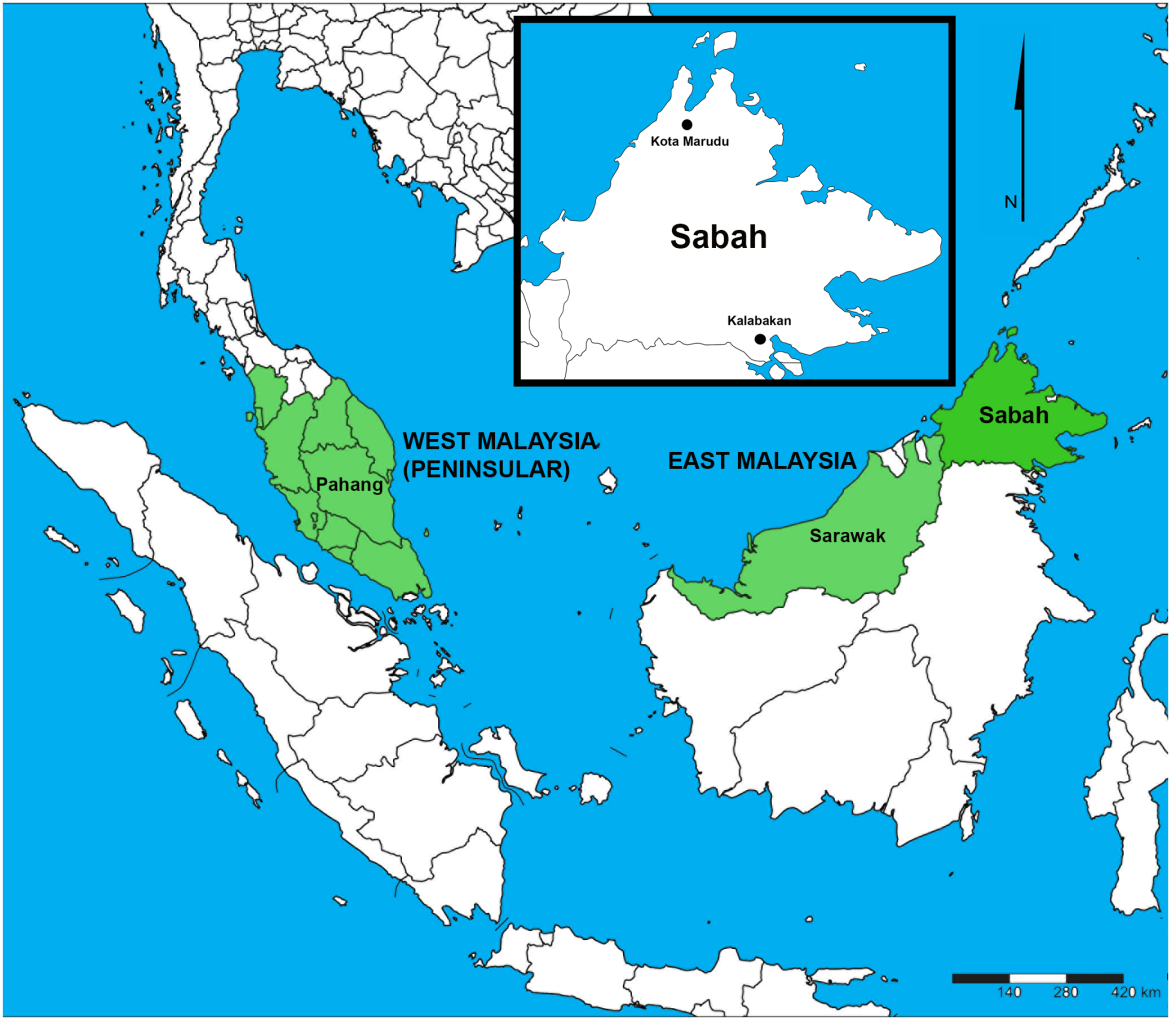
Malaria study areas in Sabah, Malaysia. In the present study, samples were collected from individuals living in two Sabah divisions from Kalabakan district in the Tawau Division and Kota Marudu district in the Kudat Division. This map was generated using SimpleMappr online software [18].

Kalabakan is located in the South of Sabah, Malaysia. Kota Marudu, is located in the North of Sabah, Malaysia (Fig 1). The study sites were chosen based on the number of malaria cases reported on the year of study (2008, 2009, 2011 and 2014) and recommendation by the Sabah State Health Department. The participants of this study were recruited from the villages, schools, palm oil and rubber plantations within these regions.

### Ethics Approval and Study Design

The ethical clearance was obtained from Research Review Committee of the Institute for Medical Research and the Medical Research Ethics Committee (MREC), Ministry of Health Malaysia. The recruited study subjects were otherwise healthy individuals from the selected sites in Kalabakan and Kota Marudu. The study subjects at the selected study sites were informed about the nature of the experiments and the procedure involved. Written informed consent was obtained from each participant or guardian before taking part in this study.

### Sample Collection

The active case detection was performed in each study site. A total of 4257 individuals were screened for malaria infection in both study areas. The *P*. *falciparum*-infected blood samples from Kalabakan were collected in 2008 and 2009. The *P*. *falciparum*-infected blood samples from Kota Marudu were collected in 2011 and 2014. Briefly, the blood samples were obtained by finger prick and malaria infection was diagnosed using rapid diagnostic test kits (Paramax-3^™^, Zephyr Biomedicals, India). Whenever a participant tested positive for *P*. *falciparum*, 50 μl of venous blood was spotted onto Whatman^®^ filter paper no. 3 (Whatman International Ltd, United Kingdom). The blood-spotted filter papers were allowed to dry completely, transferred into individual plastic bags, labeled and stored at room temperature in a dessicator containing silica gel until further processing. The malaria positive samples were also confirmed by Blood Film for Malaria Parasite technique (BFMP).

### DNA Extraction and PCR-Based Species Identification

Parasite genomic DNA was extracted from blood–spotted filter papers by using QIAmp^®^ DNA Mini Kit (QIAGEN, Germany), according to the manufacturer's instructions (dried blood spots protocol). The parasite DNA samples were kept frozen at -20°C until use. The Plasmodium species confirmation (*P*. *vivax*, *P*. *falciparum*, *P*. *ovale*, *P*. *malariae*) was done by using a technique of Padley et al. (2003) [19] with the modification that each species was diagnosed in a separate (non-multiplex) assay. The identification of *P*. *knowlesi* parasite was done by using the method of Imwong et al. (2009) [20].

### Genotyping of Antigenic Markers

*P*. *falciparum* DNA samples were further analyzed by amplification of the highly polymorphic regions of MSP-1 (Block 2), MSP-2 (Block 3) and GLURP using a set of specific primers in nested and seminested PCR method as previously described [21]. Oligonucleotide primer sets (Table 1) were used for detecting the different families of MSP-1 and MSP-2 (K1, MAD20 and RO33 in MSP-1; FC27 and 3D7 in MSP-2) and size polymorphisms of GLURP.

**Table 1:** The primers used to genotype the MSP-1, MSP-2 and GLURP polymorphic regions of *P*. *falciparum* isolates from Kalabakan and Kota Marudu, Sabah.

Gene/ Family	Primer	PCR round	Primer sequence
MSP-1	MI-OF	Primary	5’ CTAGAAGCTTTAGAAGATGCAGTATTG 3’
	MI-OR		5’ CTTAAATAGTATTCTAATTCAAGTGGATCA 3’
K1	MI-KF	Nested	5’ AAATGAAGAAGAAATTACTACAAAAGGTGC 3’
	MI-KR		5’ GCTTGCATCAGCTGGAGGGCTTGCACCAGA 3’
MAD20	MI-MF		5’ AAATGAAGGAACAAGTGGAACAGCTGTTAC 3’
	MI-MR		5’ ATCTGAAGGATTTGTACGTCTTGAATTACC 3’
RO33	MI-RF		5’ TAAAGGATGGAGCAAATACTCAAGTTGTTG 3’
	MI-RR		5’ CATCTGAAGGATTTGCAGCACCTGGAGATC 3
MSP-2	M2-OF	Primary	5' ATGAAGGTAATTAAAACATTGTCTATTATA 3'
	M2-OR		5' CTTTGTTACCATCGGTACATTCTT 3'
FC27	M2-FCF	Nested	5' AATACTAAGAGT GTAGGTGCArAT GCTCCA 3'
	M2-FCR		5' TTTTATTTGGTGCATTGCCAGAACTTGAAC 3'
3D7	M2-1CF		5' AGAAGTATGGCAGAAAGTAAkCCTyCTACT 3'
	M2-1CR		5' GATTGTAATTCGGGGGATTCAGTTTGTTCG 3'
GLURP	G-OF	Primary	5’ TGAATT GAAGATGTTCACACTGAAC 3'
	G-OR		5' GTGGAATTGCTTTTTCTTCAACACTAA 3'
	G-OR	Nested	5’ GTGGAATTGCTTTTTCTTCAACACTAA 3’
	G-NF		5’ TGTTCACACTGAACAATTAGATTTAGATCA 3’

All PCR reactions were performed using Eppendorf Mastercycler Gradient (Eppendorf, Germany). For allele detection in MSP-1 and MSP-2, PCR was done in a 50 μl PCR mixture containing 50 ng genomic DNA, 1X of MgCl_2_ free buffer, 1.5 mM of MgCl_2_, 200 μM of dNTPs, 0.2 mM of each primer and 2.5U of Taq polymerase enzyme. For GLURP allele detection, the PCR was carried out in a similar manner to the primary round of MSPs, except for using a MgCl_2_ concentration of 2.0 mM. For nested PCRs, 2 μl of primary PCR product of MSP-1 and MSP-2 and 1 μl of primary product of GLURP were used as DNA templates, which had similar reaction concentrations to the primary PCR. Cycling conditions for primary PCRs and nested PCR reactions for GLURP were as follows; the denaturation was performed at 95°C for 5 min, then followed by 25 cycles of denaturation at 94°C for 1 minute, annealing at 58°C for 45 seconds and extension at 72°C for 1.5 minutes; and a final extension was done at 72°C for 5 min. The cycling conditions for nested PCR of MSP-1 and MSP-2 started with a single step of denaturation at 95°C for 5 minutes followed by 30 cycles of denaturation at 94°C for 1 min, annealing at 61°C for 45 seconds and extension at 72°C for 1.5 minute, and a final extension at 72°C for 5 minutes.

The amplification products were analyzed using Agilent 2100 Bioanalyzer and Agilent DNA 1000 Kit (Agilent Technologies, Molecular Probes Inc, USA) conducted according to manufacturer’s instructions (S1 Fig). Alleles were considered the same if fragment sizes were within 10 bp [22] and 50 bp [23] intervals for MSPs and GLURP, respectively.

### Genotyping of Microsatellite Markers

*P*. *falciparum* DNA samples were genotyped for 10 microsatellite loci as previously described [5, 6] with slight modification. The nested PCR strategy (2 step PCR) with fluorescent end-labeled primer pairs was used for the microsatellite amplification (Table 2). Briefly, the first PCR (nested 1) was performed with the reaction mixture containing 1X PCR buffer, 2.5 mM MgCl_2_ (for ARA2, Pfg377, PfPK2, TA87, TA81, TA42 and 2490) or 3 mM MgCl_2_ (for TA1, POLYa and TA60), 125 μM dNTPs, 250 nM primer mix (F/R), 2 μl of DNA template and 0.4 U of Taq polymerase. The first PCR cycling conditions were as follows: Initial denaturation at 95°C for 5 minutes, 30 cycles of denaturing at 94°C for 1 minute, annealing at 52°C for 2 minutes, extension at 72°C for 2 minutes and last extension at 72°C for 5 minutes. One microliter of the first PCR product was used for the second PCR reaction (nested 2). The second PCR was performed with the reaction mixture containing 1 X PCR buffer, 2.5 mM MgCl_2_, 125 μM dNTPs, 250 nM primer mix (F/R), 1 μl of DNA template and 0.4 U of Taq polymerase. The second PCR cycling started with initial denaturation at 94°C for 2 minutes, followed by 5 cycles of denaturing at 94°C for 30 seconds, annealing at 50°C for 30 seconds, extension at 60°C for 30 seconds, 25 cycles of denaturing at 94°C for 30 seconds, annealing at 45°C for 30 seconds, extension at 60°C for 30 seconds and final extension at 60°C for 2 minutes. Fluorescent-labelled PCR products were sent for fragment analysis (Macrogen, Korea) and scored using Gene Mapper 5 Software (Life Technologies, USA). All electropherograms or peaks generated for each isolate were visually inspected and the predominant peaks corresponding to each allele were scored. Any samples that amplified poorly for particular loci with maximum peak height less than 200 fluorescent units were discarded. Any secondary alleles that were >30% of the height of the predominant allele peaks were also scored as additional alleles which presumably were considered to be a result of polyclonal infection.

**Table 2:** The primers used to amplify the microsatellite markers of *P*. *falciparum* isolates from Kalabakan and Kota Marudu, Sabah.

Chromosome	Locus	Primer sequence (5' to 3')	Size range (bp)
4	Poly α-R	ATCAGATAATTGTTGGTA	114–201
	Poly α-F	AAAATATAGACGAACAGA	
	Poly α-3(IR)	(HEX)GAAATTATAACTCTACCA	
12	PFPK2-3R	CCTCAGACTGAAATGCAT	159–192
	PFPK2-F	CTTTCATCGATACTACGA	
	PFPK2-R	(HEX)AAAGAAGGAACAAGCAGA	
5	TA81-3F	GAAGAAATAAGGGAAGGT	112–142
	TA81-R	TTTCACACAACACAGGATT	
	TA81-F	(HEX)TGGACAAATGGGAAAGGATA	
11	ARA2-3(F)	GTACATATGAATCACCAA	63–90
	ARA2-R	GCTTTGAGTATTATTAATA	
	ARA2-F	(HEX)GAATAAACAAAGTATTGCT	
6	TA87-3F	ATGGGTTAAATGAGGTACA	90–126
	TA87-R	ACATGTTCATATTACTCAC	
	TA87-F	(6FAM)AATGGCAACACCATTCAAC	
5	TA42-3F	ACAAAAGGGTGGTGATTCT	182–251
	TA42-R	GTATTATTACTACTACTAAAG	
	TA42-F	(6FAM)TAGAAACAGGAATGATACG	
10	2490-3R	ATGATGTGCAGATGACGA	78–93
	2490-F	TTCTAAATAGATCCAAAG	
	2490-R	(HEX)TAGAATTATTGAATGCAC	
6	TA1-3(F)	CTACATGCCTAATGAGCA	159–204
	TA1-R	TTTTATCTTCATCCCCAC	
	TA1-F	(6FAM)CCGTCATAAGTGCAGAGC	
13	TA60-F	CTCAAAGAAAAATAATTCA	69–99
	TA60-R	AAAAAGGAGGATAAATACAT	
	TA60-3(IF)	(6FAM)TAGTAACGATGTTGACAA	
12	PFG377-3R	TTATGTTGGTACCGTGTA	89–113
	PFG377-F	GATCTCAACGGAAATTAT	
	PFG377-R	(6FAM)TTATCCCTACGATTAACA	

### Data Analysis

#### Multiplicity of infection (MOI)

The multiplicity of infection (MOI) was defined as the mean number of *P*. *falciparum* genotypes per infected individual. The MOI was calculated as the proportion of the total number of *P*. *falciparum* genotypes for the same gene and the number of PCR positive isolates. Isolates with more than one genotype were considered as a polyclonal infection while the presence of a single allele was considered as monoclonal infection.

#### Allele frequency, heterozygosity and genetic differentiation analysis

To determine allele frequencies, only the predominant allele was counted for population genetic analyses. The genetic diversity in each population was assessed by calculating both the mean number of alleles (A) and the mean expected heterozygosity (*He*) across loci in each population. *He* values were calculated using the Genetic Analysis in Excel toolkit (GenAIEx) [24]. Briefly, the allelic diversity (*He*) for each microsatellite loci or antigenic markers was calculated based on the allele frequencies, using the formula $He\;=\;\left[\frac{n}{n-1}\right][1-\Sigma p^{\text{2}}]$, where n is the number of isolates analyzed and *p* represents the frequency of each different allele at a locus. *He* has a potential range from 0 (no allele diversity) to 1 (all sampled alleles are different). The genetic differentiation between populations was measured using Cockerham and Weir (1984)[25] F-Statistic analysis utilizing the Fstat software version 2.9.3.2. Genetic similarity between haplotypes was examined by creating a similarity matrix based on number of repeats of each microsatellite alleles to construct an unweighted pair group method with arithmetic mean (UPGMA) dendogram using MLVA (MLVA = Multiple Loci VNTR Analysis; VNTR = Variable Number Tandem Repeats) application in BioNumerics version 7.5 software (Applied Maths 1998–2015).

#### Analysis of multilocus linkage disequilibrium

The predominant alleles were used to construct haplotypes infection. The permutation procedure was used to test null hypothesis of random association among loci for each parasite population [26]. The number of alleles shared (*D*) between all pairwise comparisons of complete 10-locus haplotypes and the distance measure (*V*_*D*_) determination were done by using the program LIAN, version 3 [27]. To evaluate significance of deviation from random expectation (Linkage equilibrium), the observed distribution of *V*_*D*_ was compared with the distribution of *V*_*D*_ in 10,000 simulated data sets in which alleles at each locus were randomly reshuffled among genotypes. Significant linkage disequilibrium (LD) was detected if the observed *V*_*D*_ was more than 95% of the values generated in the reshuffled data sets. The standardized index of association (*I*_A_^S^) was used and calculated as *I*_A_^S^ = (*V*_*D*_*/ V*_*e*_-1)/(*r*-1), where *V*_*e*_ is the mean variance in the 10,000 reshuffled data sets, and *r* is the number of loci. The significant levels of the *I*_A_^S^ values were tested using Monte Carlo method.

## Results

### The Study Population in Kalabakan and Kota Marudu

A total of 4257 individuals were screened for malaria infection in both study areas. Specifically, 619 individuals from 23 sites in Kalabakan (2008 and 2009) were screened. Fifty eight (9.4%) tested positive for malaria of which 31 (5.0%) were positive for *P*. *falciparum*. The age of *P*. *falciparum*-infected individuals ranged from 12 to 60 years (mean age: 22.3 ± 9.3 years). The male to female sex ratio (M/F) was 1.8 (20/11). In Kota Marudu (2011 and 2014), a total of 3638 individuals from 56 sites were screened for malaria infection. Fifty four samples (1.5%) tested positive for malaria of which 29 (0.8%) were positive for *P*. *falciparum* infection. The age of *P*. *falciparum*-infected individuals ranged from 4 to 67 years (mean age: 20.3 ± 15.1 years). The male to female sex ratio (M/F) was 2.2 (20/9). The *P*. *falciparum* infected individuals were found to be blood smear-positive, asymptomatic by observation and healthy going about their routine activities. As all malaria infected individuals need to be treated in the hospital, they were advised and transported to the nearest public hospitals for malaria treatment. No clinical data was obtained in this study.

### Genotyping of MSP-1, MSP-2 and GLURP of *P*. *falciparum* Isolates in Kalabakan

Four different MSP-1 alleles were detected with 2 alleles of K1 (180 bp and 210 bp) and 1 allele of each MAD20 (180 bp) and RO33 (160 bp) allelic families. K1 allelic family was predominant in 95.6% of the *P*. *falciparum*-infected blood samples. The frequencies of monoclonal infection were 30.4% and 4.4% for K1 and RO33, respectively (Table 3). Sixty five percent of the samples were polyclonally infected (K1+ R033 and K1+MAD20) (Table 3). The MOI value for MSP-1 genotype was 1.65 (Table 3).

**Table 3:** Genotyping of *P*. *falciparum* MSP-1 polymorphic region block 2.

Study sites	MSP-1 family types	No. of samples	Allele size (bp)	Frequency (%)	MOI
Kalabakan	K1	7	180–210	30.4	1.65
	R033	1	160	4.4	
	K1 + R033	12		52.2	
	K1 + MAD20	3		13.0	
	Total	23		100.0	
Kota Marudu	K1	5	180	19.2	1.05
	MAD20	15	180–210	57.7	
	R033	5	160	19.2	
	K1 + R033	1		3.9	
	Total	26		100.0	

A total of 5 different alleles were identified in MSP-2 family genotyping (Table 4). Four alleles of 3D7/IC1 (480 bp, 570 bp, 640 bp and 660 bp) and 1 allele of FC27 were detected. The predominant allele was from the FC27 (72%) allelic family. The frequencies of monoclonal infection were 28% and 52.0% for 3D7 and FC27 allelic families, respectively (Table 4). Twenty percent of the samples were polyclonally infected (3D7 + FC27) (Table 4). The MOI value for MSP-2 genotype was 1.05 (Table 4).

**Table 4:** Genotyping of *P*. *falciparum* MSP-2 polymorphic region block 3.

Study sites	MSP-2 family types	No. of samples	Allele size (bp)	Frequency (%)	MOI
Kalabakan	3D7	7	480–660	28.0	1.20
	FC27	13	300–310	52.0	
	3D7 + FC27	5		20.0	
	Total	25		100.0	
Kota Marudu	3D7	18	520	81.8	1.05
	FC27	3	300–340	13.6	
	3D7 + FC27	1		4.6	
	Total	22		100.0	

Twenty four samples were successfully genotyped for RII repeat region of GLURP (Table 5). Five groups of GLURP genotypes were detected in Kalabakan *P*. *falciparum*-infected blood samples ranging from 551 bp to 800 bp (50 bp bin) and coded as genotypes II-VI (Table 5). Only 1 allele was detected in all individual samples suggesting a monoclonal infection. Frequency of genotype VI was the highest (41.6%) followed by genotype V (29.2%) and genotype IV (20.8%). Only one sample was found in genotype II (4.2%) and genotype III (4.2%) (Table 5). Genotype I was not detected in Kalabakan *P*. *falciparum*-infected samples.

**Table 5:** Distribution of allelic variants of GLURP RII repeat region of *P*. *falciparum* populations in Kalabakan and Kota Marudu.

Genotypes	Allelic size variants (50 bp bin)	Kalabakan (N = 24), N (%)	Kota Marudu (N = 28), N (%)
I	501–550	0 (0)	2 (7.1)
II	551–600	1 (4.2)	0 (0)
III	601–650	1 (4.2)	5 (17.9)
IV	651–700	5 (20.8)	14 (50.0)
V	701–750	7 (29.2)	6 (21.4)
VI	751–800	10 (41.6)	1 (3.6)

N, number of sample.

### Genotyping of MSP-1, MSP-2 and GLURP of *P*. *falciparum* Isolates in Kota Marudu

Five different alleles were detected in MSP-1 family typing. These alleles comprised of 1 allele of K1 (180 bp), 3 alleles of MAD20 (180 bp, 200 bp and 210 bp) and 1 allele of R033 (160 bp) allelic families. The MAD20 was predominant (57.7%) in Kota Marudu *P*. *falciparum*-infected blood samples (Table 3). The frequencies of monoclonal infection were 57.7% for MAD20 and 19.2% for both K1 and R033 allelic families (Table 3). Only 1 sample (3.9%) was polyclonally infected (K1 + R033) (Table 3). The MOI value for MSP-1 genotype was 1.05 (Table 3).

A total of three different alleles were detected in MSP-2 family typing. Only 1 allele belongs to 3D7/IC1family and 2 alleles belong to FC27 family. The predominant MSP-2 allele family in Kota Marudu *P*. *falciparum*-infected blood samples was 3D7 (86.4%). The frequencies of monoclonal infection were 81.8% and 13.6% for both 3D7 and FC27 allelic families, respectively (Table 4). Only 1 sample (4.6%) was polyclonally infected (3D7 + FC27) (Table 4). The MOI value for MSP-2 genotype was 1.05 (Table 4).

Twenty eight samples were successfully genotyped for GLURP RII repeat region (Table 5). Five GLURP genotypes were detected in Kota Marudu *P*. *falciparum*-infected blood samples ranging from 501 bp to 800 bp (50 bp bin) and coded as genotype I, III, IV, V and VI. Similar to Kalabakan, only 1 allele was detected in all Kota Marudu *P*. *falciparum*-infected blood samples. Frequency of genotype IV was the highest (50.0%) followed by genotypes V (21.4%), III (17.9%), I (7.1%) and VI (3.6%) (Table 5). Genotype II was not detected in Kota Marudu samples.

### Microsatellites Genotyping of Kalabakan and Kota Marudu *P*. *falciparum* Isolates

**Fig 2:**
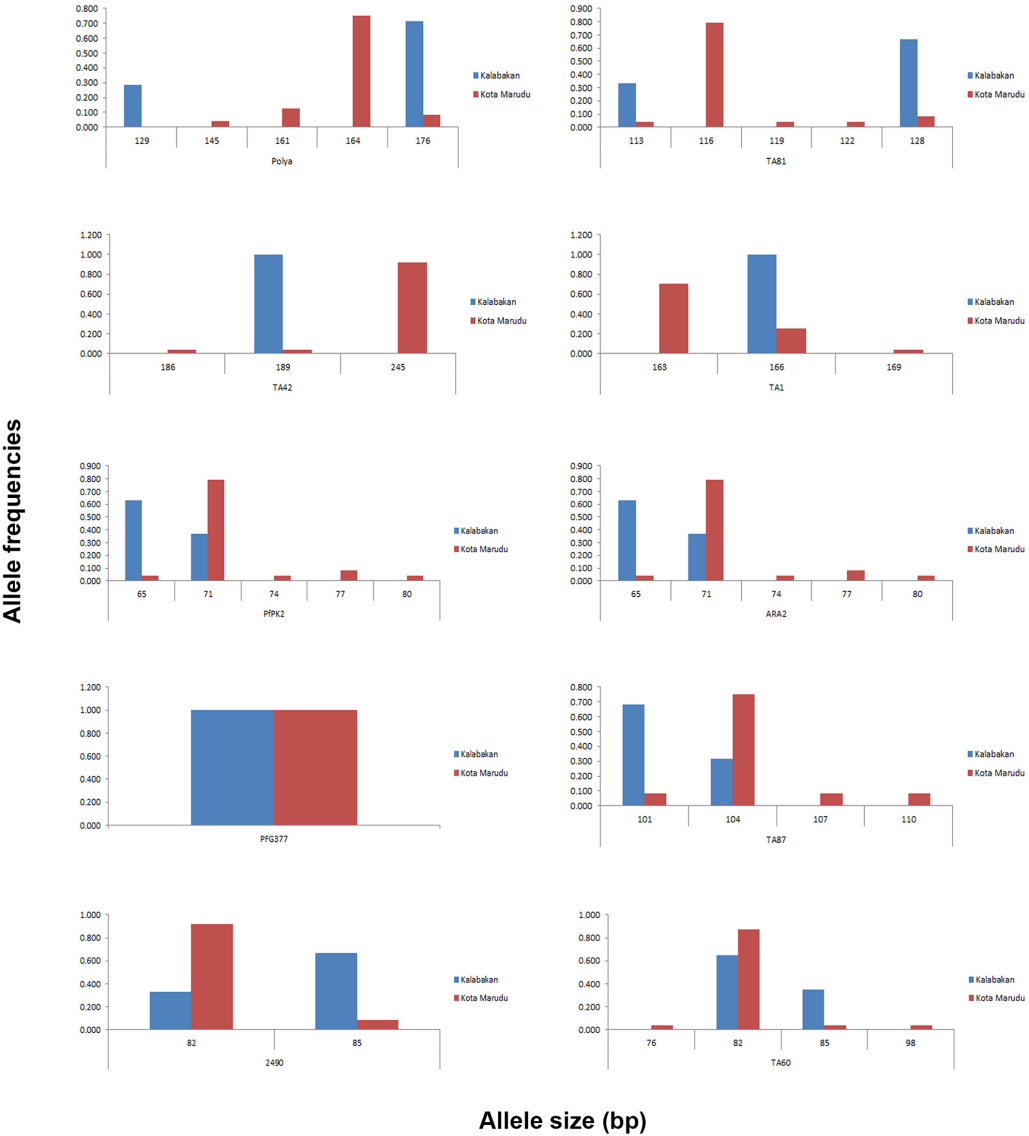
The allele frequencies of 10 microsatellite loci in the Kalabakan and Kota Marudu *P*. *falciparum* isolates. Allele sizes, determined by fragment analysis, are shown on the x-axis and their frequencies on the y-axis. Vertical bars for allele frequencies of each microsatellite locus were determined using GENALEX 6.0. Only 1 allele (96 bp) was detected on PFG377 locus for both Kalabakan and Kota Marudu *P*. *falciparum* isolates (S1 Table).

The Kalabakan datasets comprised of multilocus microsatellite alleles derived from 14 to 19 *P*. *falciparum*-infected blood samples. The allele frequencies of each microsatellite locus were shown in Fig 2 and S1 Table. Number of alleles per locus in the total Kalabakan sample ranged from 1 to 2 (Table 6). There was no polyclonal infection observed in any of the samples.

**Table 6:** Distribution of 10 microsatellite loci of *P*. *falciparum* isolates in Kalabakan and Kota Marudu.

Sites	Number of samples (N)	Locus	Allele size range (bp)	No. of Alleles	Expected heterozygosity (*He*)
Kalabakan	14	POLYa	129–176	2	0.44
	18	TA81	113–128	2	0.47
	18	TA42	189	1	0.00
	17	TA1	166	1	0.00
	19	PfPK2	65–71	2	0.49
	19	ARA2	65–71	2	0.49
	18	PFG377	96	1	0.00
	19	TA87	101–104	2	0.46
	18	2490	82–85	2	0.47
	17	TA60	82–85	2	0.49
		Mean ± SE		1.70 ± 0.15	0.33 ± 0.07
Kota Marudu	24	POLYa	145–176	4	0.43
	24	TA81	113–128	5	0.38
	24	TA42	186–245	3	0.16
	24	TA1	163–169	3	0.45
	24	PfPK2	65–80	5	0.38
	24	ARA2	65–80	5	0.38
	24	PFG377	96	1	0.00
	24	TA87	101–110	4	0.44
	24	2490	82–85	2	0.16
	24	TA60	76–98	4	0.24
		Mean ± SE		3.60 ± 0.43	0.30 ± 0.05

In Kota Marudu, twenty-four *P*. *falciparum*-infected samples were successfully amplified for all 10 microsatelite loci. The allele frequencies for each microsatellite marker are shown in Fig 2 and S1 Table. Number of alleles per locus ranged from 1 to 5 (Table 6). Similar to Kalabakan *P*. *falciparum*-infected blood samples, there was no polyclonal infection observed in any of the samples.

Except PFG377, all locus showed the distribution of major alleles with high frequency (Fig 2). The major alleles in each locus were found to be unique for each Kalabakan and Kota Marudu *P*. *falciparum*-infected blood samples (Fig 2). The mean number of alleles per locus for Kalabakan and Kota Marudu *P*. *falciparum-*infected blood samples were 1.70 and 3.60, respectively (Table 6).

### Genetic Diversity Analysis of *P*. *falciparum* Populations in Kalabakan and Kota Marudu

The number of microsatellite haplotypes were examined and samples with any missing genotypes or greater than two alleles at any locus were excluded. Overall, 10 haplotypes were identified in thirty nine collective Kalabakan and Kota Marudu *P*. *falciparum*-infected blood samples and were grouped alphabetically from A to J (S2 Table). Each haplotype was varied at either one or more locus. The *P*. *falciparum* population in Kalabakan was predominated with haplotype B (62.5%) followed by haplotype A (37.5%). Both haplotypes were present in *P*. *falciparum*-infected blood samples collected in 2008 and 2009. In contrast, the major haplotype in Kota Marudu was haplotype C (62.5%) followed by haplotype D (8.33%), B (4.17%), E (4.17%), F (4.17%), G (4.17%), H (4.17%), I (4.17%) and J (4.17%). Except haplotype D, all haplotypes were present in *P*. *falciparum*-infected blood samples collected in year 2011. However, only haplotype C (75%) and D (25%) appeared in *P*. *falciparum*-infected blood samples collected in year 2014 (S2 Fig).

Low level of genetic diversity was detected for both Kalabakan and Kota Marudu *P*. *falciparum* populations. In Kalabakan, the expected heterozygosity (*He*) values for each microsatellite locus ranged from 0 to 0.49 (Table 6). The similar range of *He* values were also detected for Kota Marudu *P*. *falciparum* population which ranged from 0 to 0.45 (Table 6). The mean microsatellite *He* values for each Kalabakan and Kota Marudu *P*. *falciparum* populations were 0.33 and 0.30, respectively (Table 6). The *He* level was also determined for antigenic markers, MSP-1, MSP-2 and GLURP. In Kalabakan *P*. *falciparum* population, low level of diversity was observed for MSP-1 and MSP-2 with *He* values of 0.17 and 0.37, respectively (Table 7). The intermediate level of diversity was observed for GLURP with *He* value of 0.70 (Table 7). The low diversity level was also observed in Kota Marudu *P*. *falciparum* population with *He* value of 0.24 and 0.25 for MSP-1 and MSP-2, respectively (Table 7). Similar to Kalabakan *P*. *falciparum* population, the intermediate level of diversity was scored on GLURP antigen with *He* value of 0.69 (Table 7). The average *He* values (±SEM) were 0.35 (±0.06) and 0.33 (±0.05) for Kalabakan and Kota Marudu *P*. *falciparum* populations, respectively.

**Table 7:** The genetic diversity of *P*. *falciparum* populations by each neutral microsatellite and antigenic markers.

Sites		Microsatellites										Antigens		
		PfPK2	TA42	TA1	TA81	ARA2	POLYa	PFG377	TA87	2490	TA60	MSP-1	MSP-2	GLURP
**Kalabakan**	**N**	19	18	17	18	19	14	18	19	18	17.0	13	15	24
	***Na***	2	1	1	2	2	2	1	2	2	2.0	1.3	3.0	5
	***He***	0.491	0.000	0.000	0.471	0.491	0.440	0.000	0.456	0.471	0.485	0.173	0.374	0.703
**Kota Marudu**	**N**	24	24	24	24	24	24	24	24	24	24	8	12	28
	***Na***	5	3	3	5	5	4	1	4	2	4	1.7	1.5	5
	***He***	0.377	0.163	0.453	0.377	0.377	0.431	0.000	0.435	0.159	0.239	0.242	0.250	0.690

N, number of sample; *Na*, number of allele; *He*, expected heterozygosity

### Linkage Disequilibrium and Genetic Differentiation

All blood samples collected in Kalabakan and Kota Marudu were found to be monoclonally infected with *P*. *falciparum* parasite. Therefore, all of the *P*. *falciparum*-infected blood samples were included in linkage disequilibrium (LD) determination. To test the significance of LD, the Monte Carlo method [26] was used. Significant LD (*p*<0.01) was found in both Kalabakan and Kota Marudu *P*. *falciparum* populations with *I*^*A*^_*S*_ values of 0.495 and 0.601, respectively (Table 8).

**Table 8:** Multilocus linkage disequilibrium and genetic differentiation between Kalabakan and Kota Marudu *P*. *falciparum* populations.

Sites	N	LD (*I*^*A*^_*S*_)	F_ST_
Kalabakan	17.70 ± 0.47	0.495^a^	0.532^b^
Kota Marudu	24.00 ± 0.00	0.601^a^	

***I***_***A***_^***S***^, index of association; N, number of sample; F_ST_, F statistic; LD, linkage disequilibrium.

^a^ Significant value, p<0.01.

^b^Significant value, p<0.05.

**Fig 3:**
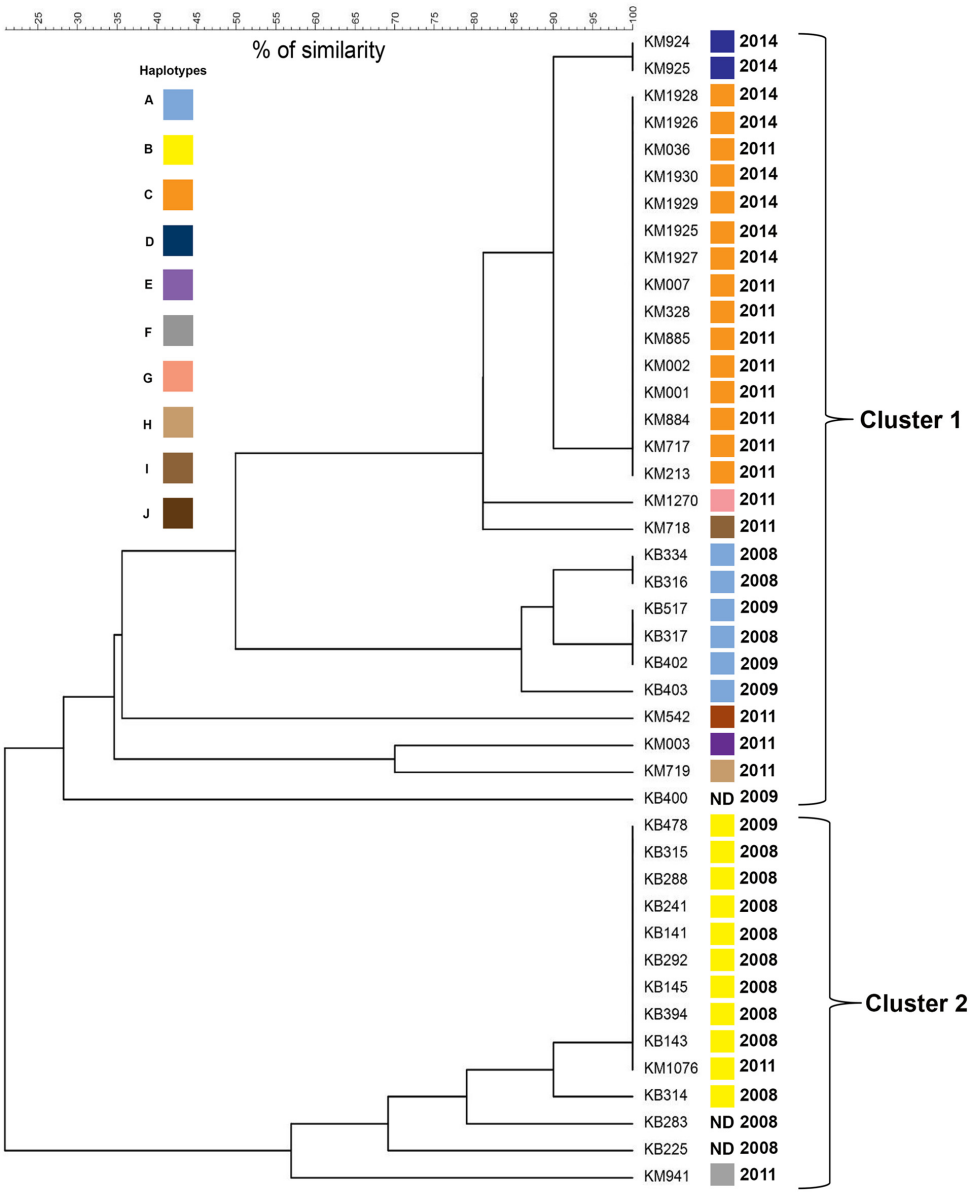
Dendogram showing the clustering of 43 *P*. *falciparum* isolates from Kalabakan (KB) and Kota Marudu (KM). The genetic relatedness between microsatellite haplotypes was examined by generating a similarity matrix based on number of tri-nucleotide repeats of each allele from 10 microsatellite loci to construct an unweighted pair group method with arithmetic mean (UPGMA) dendogram. The color coded haplotypes show the distribution of different clonal *P*. *falciparum* isolates within each cluster. The major haplotypes (A, B and C) persisted in every year of sampling (2008 and 2009 for Kalabakan; 2011 and 2012 for Kota Marudu). ND, the haplotype was not determined due to missing genotypes or more than 2 alleles of any microsatellite locus.

The approximate road distance between Kalabakan and Kota Marudu was 486 km. For genetic differentiation analysis, the F_ST_ [25] was calculated. A significant genetic differentiation was observed between Kalabakan and Kota Marudu *P*. *falciparum* populations with F_ST_ value of 0.532 (p<0.05). The genetic relatedness between Kalabakan and Kota Marudu *P*. *falciparum* populations was illustrated in UPGMA dendogram in Fig 3. Based on the number of microsatellite repeats, the *P*. *falciparum* isolates were obviously grouped into 2 major clusters at 20% of similarity, cluster A and B, where each of the cluster was predominated with Kota Marudu and Kalabakan *P*. *falciparum* populations, respectively (Fig 3).

## Discussion

The *P*. *falciparum* malaria risk index in Sabah is classified as low stable transmission with an annual parasite incidence (API) of ≥ 0.1 to < 1.0 case per 1000 population [1]. Outdoor malaria transmission remains a major problem in isolated indigenous populations living in forest or forest-fringe areas. The vector ecology and transmission patterns of malaria in these areas present a unique challenge for vector control program. The populations in these areas do not generally have the same level of access to health care as the rest of the population due to the mountainous nature of these areas and lack of proper terrain for transportation. Many of these indigenous people also depends on jungle resources and night time activities such as hunting for their daily living, hence exposing themselves to malaria infection through mosquito bites. In addition, deforestation has also contributed to the transmission of the disease largely as result of outdoor laborers and jungle workers exposure to the vectors. These reasons justified the choice of isolated forest settlements or villages and plantation camps as the sites for the present study.

The evidence of *P*. *falciparum* populations with limited diversity in other areas of Sabah exist even before the implementation of National Malaria Elimination Program in 2011 (sample collection between year 1997 to 2003) [11]. This suggest the current *P*. *falciparum* population structure was created by the impact of the previous malaria control interventions in the region. The genetic diversity analysis in the present study showed that *P*. *falciparum* populations in Kalabakan and Kota Marudu exhibited an overall low level of diversity (*He*). In addition, the occurrence of major microsatellite haplotypes in Kalabakan (2008–2009) and Kota Marudu (2011–2014) seem to be persisted in every sampling periods (Fig 3; S2 Table; S2 Fig). Other areas of Sabah like Banggi Island, Telupid, Lahad Datu, Kunak and Malinsau were previously reported with a low level of *He* (Table 9) and all except Malinsau, exhibited a significant LD within each population [11]. Similarly, the present study showed that both Kalabakan and Kota Marudu *P*. *falciparum* populations also exhibited a significant LD (*I*^*A*^_*S*_ = 0.495 and 0.601, respectively) (Table 8) suggesting a high selfing rate between parasites during the sexual stages of mosquito transmission, resulting in a largely clonal parasite population. The domination of clonal *P*. *falciparum* population is expected to be observed in regions with declining endemicity as the result of genetic drift caused by scale up interventions [10]. In other words, the persistence of a particular haplotype throughout the year is expected in parasite populations where control efforts have left behind lineages that create the clusters of highly related parasites [10, 28]. Thus, unrelated parasites are unlikely to occur together in the same mosquito blood meal. Other countries or regions with declining malaria transmission such as Djibouti [9], Colombia [29], Venezuela [28], Peru [30], Thailand [3, 12, 31], some parts of Philippines [13] and Indonesia [32] have also been reported to have malaria infections with low level of *He*, MOI and LD (Table 9).

**Table 9:** The diversity level of *P*. *falciparum* populations in different transmission settings.

Countries/Locations	Data	Mean MOI	*He*	LD (*I*^*A*^_*S*_)	Transmission status (Program phase)^a^	Study
**Malaysia**					Foci (Pre-elimination)	
Sabah, East Malaysia						
*Malinsau*	Microsatellites	*-*	0.63	0.009		Anthony et al. 2005 [11]
*Telupid*	Microsatellites	*-*	0.48	0.190		
*Lahad Datu*	Microsatellites	*-*	0.55	0.118		
*Kunak*	Microsatellites	*-*	0.46	0.104		
*Kalabakan*	Microsatellites	*-*	0.33	0.495		Present study
*Kota Marudu*	Microsatellites	*-*	0.30	0.601		
*Kalabakan*	MSP-1, MSP-2 & GLURP	1.28	0.17–0.70	-		Present study
*Kota Marudu*	MSP-1, MSP-2 & GLURP	1.03	0.24–0.70	-		
Sarawak, East Malaysia						
*Lundu*	Microsatellites	*-*	0.44	0.022		Anthony et al. 2005 [11]
*Bau*	Microsatellites	*-*	0.47	0.149		
*Serian*	Microsatellites	*-*	0.50	0.075		
Pahang, Peninsular Malaysia	MSP-1 & MSP-2		0.55–0.57	-		Atroosh et al. 2011 [17]
**Thailand**					Low (Control)	
*Maehongson*	Microsatellites	*-*	0.68	-		Pumpaibool et al. 2009 [12]
*Tak*	Microsatellites	*-*	0.63	-		
*Kanchanaburi*	Microsatellites	*-*	0.60	-		
*Ubonratchathani*	Microsatellites	*-*	0.70	-		
*Trat*	Microsatellites	*-*	0.56	-		
*Ranong*	Microsatellites	*-*	0.62	-		
*Yala*	Microsatellites	*-*	0.00	-		
*Ranong*	MSP-1, MSP-2 & GLURP	3.47	-	-		Congpuong et al. 2014 [31]
*Kanchanaburi*	MSP-1, MSP-2 & GLURP	3.15	-	-		
*Tak*	MSP-1, MSP-2 & GLURP	3.14	-	-		
	SNPs	*-*	0.41–0.42	0.003–0.011		Nkhoma et al. 2013 [3]
**Indonesia**					Low (Control)	
*Papua*	MSP-1, MSP-2 & GLURP	6.00–10.00	-	-		Sulistyaningsih et al. 2013 [33]
*South Kalimantan*	MSP-1, MSP-2 & GLURP	2.00–7.00	-	-		
*South Kalimantan (Ketapang)*	Microsatellites	1.00	0.40	0.239		Noviyanti et al. 2015 [32]
*Bangka*	Microsatellites	1.14	0.46	0.048		
*Sumba*	Microsatellites	1.23	0.72	0.018		
*West Timor*	Microsatellites	1.11	0.52	0.229		
**Philipines**					Low (Control)	
*Kalinga*	Microsatellites	-	0.39	0.104		Iwagami et al. 2009 [13]
*Palawan*	Microsatellites	-	0.60	0.043		
*Davao del Norte*	Microsatellites	-	0.51	0.052		
**South American**						
*Venezuela*	Microsatellites	*-*	0.39	0.172	Low (Control)	Chenet et al. 2012 [28]
*Colombia*	Microsatellites	5.00	0.18	0.163	Low (Control)	Chenet et al. 2015 [29]
**African countries**						
*Malawi*	MSP-1, MSP-2 & GLURP	1.01–1.52	0.89–0.97	-	High (Control)	Mwingira et al. 2011 [23]
*Burkina Faso*	MSP-1, MSP-2 & GLURP	1.40–3.03	0.78–0.98	-	High (Control)	
*Sao Tome*	MSP-1, MSP-2 & GLURP	1.01–2.00	0.83–0.96	-	High (Control)	
*Tanzania*	MSP-1, MSP-2 & GLURP	1.84–3.48	0.84–0.99	-	High (Control)	
*Uganda*	MSP-1, MSP-2 & GLURP	1.17–1.29	0.68–0.95	-	High (Control)	
*Papua New Guinea*	Microsatellites	-	0.79	0.009	High (Control)	Schultz et al. 2010 [34]
	MSP-2	1.00–2.12	0.90–0.96	-		Barry et al. 2013 [35]
*Guinea*	Microsatellites	3.7–4.2	0.77–0.79	0.032–0.106	High (Control)	Mobegi et al. 2012 [16]
*Guinea Bissau*	Microsatellites	2.60	0.80	0.000	High (Control)	
*Gambia*	Microsatellites	1.7–2.4	0.75–0.76	0.007–0.017	High (Control)	
*Senegal*	Microsatellites	2.20	0.72	0.150	High (Control)	
*Nigeria*	Microsatellites	1.64–1.79	0.65–0.79	0.021–0.062	High (Control)	Oyebola et al. 2014 [15]
	MSP-1 & MSP-2	1.39–1.76	-	-		Oyebola et al. 2014 [14]
*Djibouti*	Microsatellites	1.00	0.00	-	Low (Control)	Khaireh et al. 2013 [9]

MOI, multiplicity of infection; *He*, expected heterozygosity; *I*_*A*_^*S*^, index of association; LD, linkage disequilibrium.

^a^ Based on the WHO Malaria Country Profile 2015 [1].

The scale up interventions such as the usage of insecticide-treated bed nets, indoor residual spray [4, 36] and the introduction of new antimalarial drug regimens [3, 28–30, 37–40] to control and treat malaria have been shown to cause the genetic drift and decrease the level of *He* and MOI. It is possible that previous implementation of antimalarial drug policies in Malaysia such as the use of chloroquine (1960s) and pyrimethamine-sulfadoxine combination therapy (1970s) may have caused the selection of particular *P*. *falciparum* genotype. Indeed, the Kalabakan *P*. *falciparum* populations with chloroquine (81%) [41], pyrimethamine (52%) and sulfadoxine (74%) resistant genotypes have been reported [42]. High prevalence of pyrimethamine-sulfadoxine resistant *P*. *falciparum* has also been reported in other parts of Sabah [43]. In addition, high percentage of *P*. *falciparum* isolates carrying the chloroquine resistance genotype were also reported in low transmission area in Peninsular Malaysia like Pahang [44]. Characterization of the parasite population using a combination of neutral microsatellite markers and loci linked to known drug resistance mutations will lead to a better understanding on the drug selection effect [29].

High transmission regions like those occurring in many African countries are normally characterized by *P*. *falciparum* populations that are genetically diverse. Antigenic marker genotyping carried out in African regions like Burkina Faso, Sao Tome, Malawi, Uganda and Tanzania have identified *P*. *falciparum* populations with alleles occurring at a frequency below than 10 percent with a very high *He* level (0.78 to 0.99) [23] (Table 9). Microsatellites analysis also revealed *P*. *falciparum* populations in African regions (except Djibouti) exhibited a high level of *He* (0.72 to 0.80) and low LD [15, 16] (Table 9). A similar situation also occurs in a foci of high levels of human migrations, like at the Thai-Myanmar border (Tak, Kanchanaburi and Ranong) where the majority (72.9%) of the *P*. *falciparum* populations showed MOI of more than 3 [31] (Table 9).

Frequent migration of humans within sub-regions of the continent, vector transmission intensity, large gametocyte reservoir, antimalarial drug resistance and lack of combined and intensive prevention and intervention measures have historically intensified the level of *P*. *falciparum* diversity in most African countries. Moreover, circulating multiple clone parasites can be ingested by the mosquitos, hence, increasing the level of outbreeding in these regions [5, 10, 45]. This situation has proved challenging for malaria control efforts. Scale-up intervention such as the implementation of insecticide-treated bed nets and ACT treatment in Western Kenya failed to reduce the level of *P*. *falciparum* diversity [36, 46]. Similarly, implementation of a comprehensive malaria control program involving indoor residual spraying, universal coverage with ITN, use of rapid diagnostic tests and treatment with ACT did not caused any changes in the proportion of multiple-genotype infections, *He*, LD, genotypic richness and effective population size in Malawi [47]. However, it has to be noted that such changes in genetic diversity may not be seen within short period of time and other factors such as frequent gene flow could hinder the intervention effects in high transmission area [36, 47].

The fragmentation of population structure due to large genetic variation or contrasting genetic patterns between parasite populations is expected in the malaria declining areas [10, 11]. Indeed, each of Kalabakan and Kota Marudu *P*. *falciparum* population were carrying different predominant family genotype of MSP-1 and MSP-2 (K1 and FC27 for Kalabakan population and MAD20 and 3D7 for Kota Marudu population). This followed by predomination of GLURP VI and GLURP IV genotypes in Kalabakan and Kota Marudu *P*. *falciparum* populations, respectively. Moreover, the different pattern of major microsatellite haplotypes were also observed in Kalabakan and Kota Marudu *P*. *falciparum* populations (Fig 2; S2 Table). With approximate road distance of 486 km, the F^ST^ value between Kalabakan and Kota Marudu reached 0.532 (Table 8) suggesting a great genetic differentiation. The geographical distance between the areas could limit the frequency of gene flow. These findings are also in line with a previous study conducted by Anthony et al. (2005) which highlighted the occurrence of contrasting population structure and low level of gene flow between many of the *P*. *falciparum* populations within East Malaysia where a strong relationship between genetic differentiation and geographical distance is observed [11]. Another contributing factor could be the successful implementation of integrated vector control management and prompt malaria treatment such as the use of ACT in the current Ministry of Health policy, hence preventing the spread and importation of malaria. In contrast, the low level of genetic differentiation reported between *P*. *falciparum* populations in high transmission setting regions like African countries where frequent mosquito biting and active human migration between continents coexist. This is normally supported by insignificant overall evidence for isolation by distance [5, 15, 16]. Further comparison of genetic differentiation levels between regions with different transmission settings were shown in Table 10.

**Table 10:** Interpopulation comparison of F_ST_ between *P*. *falciparum* from Asian and other countries with different transmission intensity.

Countries (Transmission intensity)	Locations compared	F_ST_	Study
Sabah, Malaysia (Foci)	Banggi Island/Telupid	0.230	Anthony et al. 2005 [11]
	Lahad Datu/Malinsau	0.175	
	Kalabakan/Kota Marudu	0.532	Present study
Philipines (Low)	Palawan/Kalinga	0.096	Iwagami et al. 2009 [13]
	Palawan/Mindanao Island	0.101	
	Mindanao Island/Kalinga	0.144	
Thailand (Low)	Maehongson/Kanchanaburi	0.037	Pumpaibool et al. 2009 [12]
	Tak/Kanchanaburi	0.015	
	Kanchanaburi/Yala	0.619	
	Tak/Yala	0.559	
West African (High)	N'Zerekore/Basse	0.019	Mobegi et al. 2012 [16]
	N'Zerekore/Boke	0.009	
	Boke/Basse	0.021	
	Basse/Forecariah	0.012	
	N'Zerekore/Farafenni	0.007	

F_ST_, F statistic

Understanding the population structure and diversity of *P*. *falciparum* has implications for vaccine, drug, and epidemiological studies including monitoring malaria during and after the elimination phase [8, 28–30, 39, 48]. Other interventions such as the introduction and regulation on the use of ACT for malaria chemotherapy in 2008 may further decreased the genetic diversity of malaria parasite populations in Malaysia. Therefore, continuous malaria genetic surveillance is important in monitoring the scale up intervention effectiveness, especially in the pre-elimination phase regions. However, monitoring of antimalarial drug efficacy with current genotyping approaches might be challenging due to the possibility of reinfection by highly related parasites being difficult to distinguish from true parasite recrudescence due to drug failure [49–51]. The potential solution to this problem is to emphasize the importance of PCR correction, improved study design and statistical methods to compensate for misclassification, to develop a highly sensitive and improved genotyping method and to conduct trials in non-endemic regions or isolate patients to prevent the chance of re-infection [50].

The limitation of the present study is the small sample size collected by active case detection. As the pre-elimination phase region, the number of malaria cases in Malaysia particularly Sabah decrease drastically each year, only a small amount of *P*. *falciparum* infected sample could be collected within the study period after screening of 4257 individuals. However, the present study has robustly genotyped the *P*. *falciparum* isolates on 3 antigenic (MSP-1, MSP-2 and GLURP) and 10 neutral microsatellite markers with efforts to obtain a conclusive genetic diversity dataset for the areas. Although the malaria positive individuals were advised and transported to the nearest hospital for treatment, the information on clinical data could not be obtained during the study. Thus, the relationship between malaria severity or clinical symptom and genetic diversity could not be addressed in the present study. The seasonal or yearly collection of sample by passive case detection will give more information on the malaria clinical symptom and severity that may represent the population structure of *P*. *falciparum* in the study areas during the elimination process. Despite these limitations, the data from the present study has confirmed the limited genetic diversity profile and contrasting population structure of *P*. *falciparum* populations in Kalabakan and Kota Marudu, key remaining malaria residual foci in Sabah.

## Conclusions

The study presented baseline genetic diversity data of *P*. *falciparum* isolates in remaining malaria residual foci areas of Kota Marudu and Kalabakan, Sabah prior to the targeted elimination of malaria by 2020. Low level of genetic diversity and predomination of clonal *P*. *falciparum* populations reflected the decline of malaria transmission in the study areas. However, the clonal characteristic of circulating *P*. *falciparum* populations will present a challenge in drug efficacy trials, as current genotyping technique may lead to false recrudescence data which could in turn have a profound impact on policy decisions.

## Supporting Information

S1 Fig The representative of electrophoresis and genotyping analysis by using bioanalyzer. (PDF)

S2 Fig Divergence between Kalabakan and Kota Marudu *P*. *falciparum* population structure and the persistence of major genotypes over time based on the distribution of microsatellite haplotype constructs. (PDF)

S1 Table Allele frequencies of each 10 *P*. *falciparum* microsatellite locus. (PDF)

S2 Table Microsatellite haplotype constructs of Kalabakan and Kota Marudu samples by year. (PDF)
