# Supplementary material for: Genetic Diversity of Plasmodium falciparum Populations in Malaria Declining Areas of Sabah, East Malaysia
Source: PLoS One. 2016 Mar 29;11(3):e0152415. doi: 10.1371/journal.pone.0152415 (PMC4811561; doi:10.1371/journal.pone.0152415)
Supplement: S1 Fig — (PDF) [file pone.0152415.s001.pdf]

A) MSP-1: Family K1 and MAD20

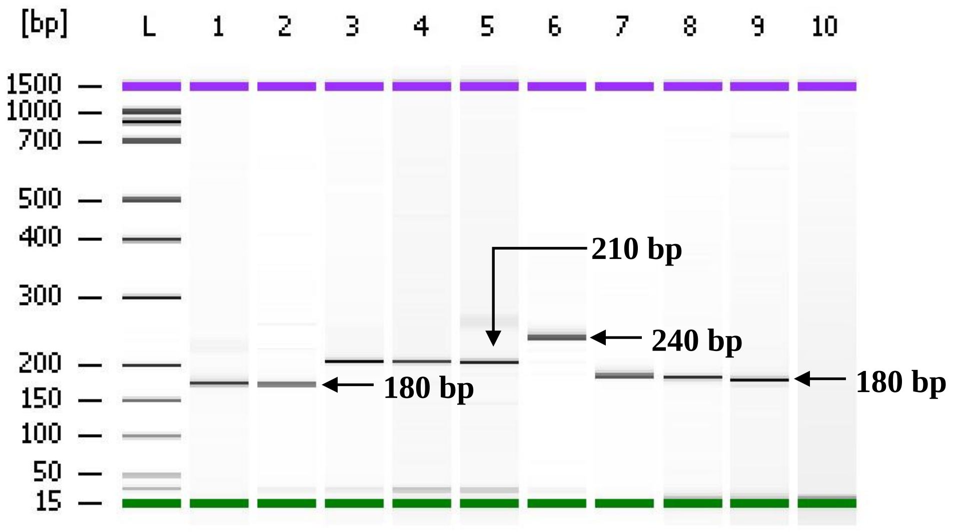

B) MSP-1: Family RO33

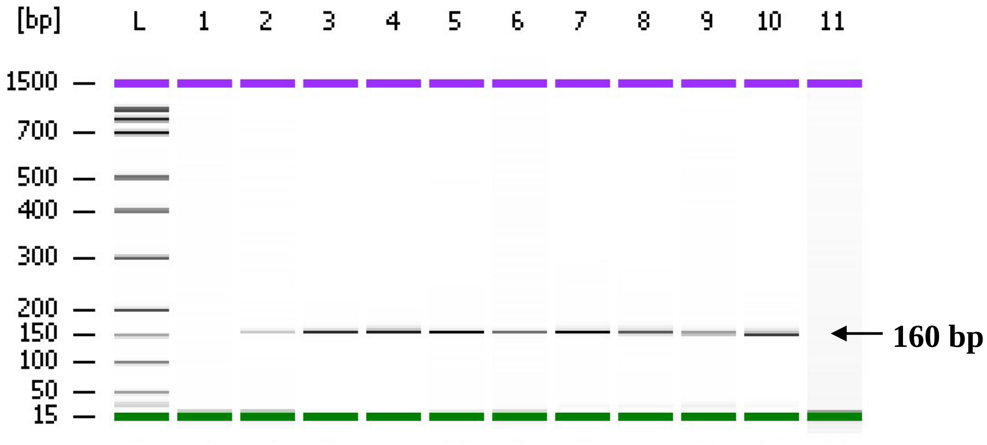

C) MSP-2: Family 3D7

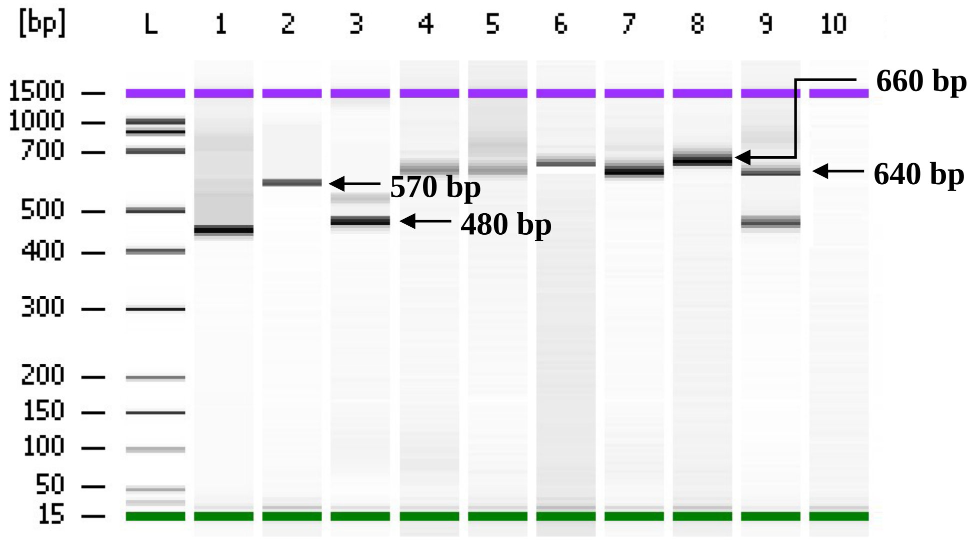

D) MSP-2: Family FC27

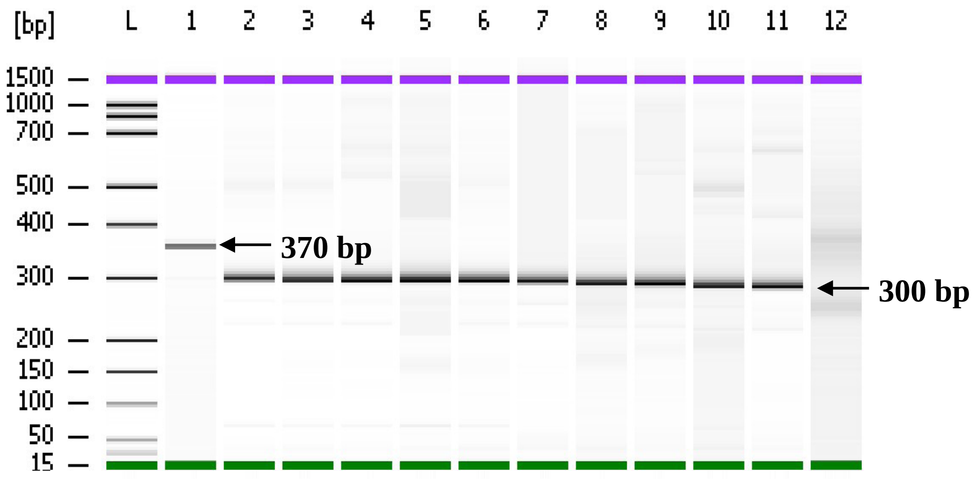

E) GLURP

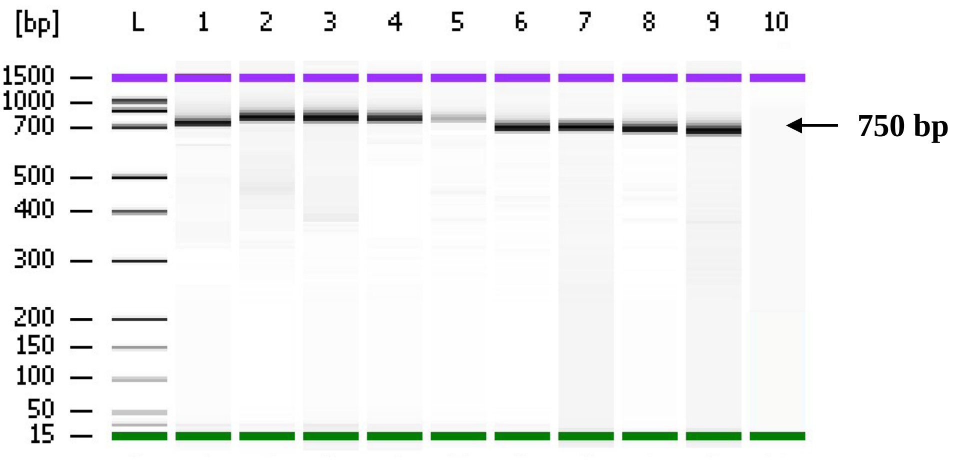

S1 Fig. The representative of electrophoresis and genotyping analysis by using bioanalyzer. (A) PCR with primers MI-KF/MI-KR for K1 (well 1-5) and MI-MF/MI-MR for MAD20 (well 6-10). L: DNA marker; well 1: *P. falciparum* K1 strain (positive control); well 2-4, 7-8: field samples; telaga 5: strain *P. falciparum* T9.96 (positive); well 6: *P. falciparum* 3D7 strain (negative); well 10: PCR negative control. (B) PCR with primers MI-RF/MI-RR for R033. L: DNA marker; well 1: *P. falciparum* K1 strain (negative control); well 2-10: field samples; well 11: PCR negative control. (C) PCR with primers M2-ICF/M2-ICR for 3D7. L: DNA marker; well 1 and 2: *P. falciparum* 3D7 and K1 strain (positive control); well 3-9: field samples; well 10: PCR negative control. (D) PCR with primers M2-FCF/M2-FCR for FC27. L: DNA marker; well 1 *P. falciparum* K1 strain (positive control); well 2-11: field samples; well 12: PCR negative control. (E) PCR with primers G-NF/G-OR for GLURP. L: DNA marker; well 1 *P. falciparum* 3D strain (positive control); well 2-9: field samples; well 10: PCR negative control.
