# Supplementary material for: Genetic Diversity of Plasmodium falciparum Populations in Malaria Declining Areas of Sabah, East Malaysia
Source: PLoS One. 2016 Mar 29;11(3):e0152415. doi: 10.1371/journal.pone.0152415 (PMC4811561; doi:10.1371/journal.pone.0152415)
Supplement: S2 Fig — (PDF) [file pone.0152415.s002.pdf]

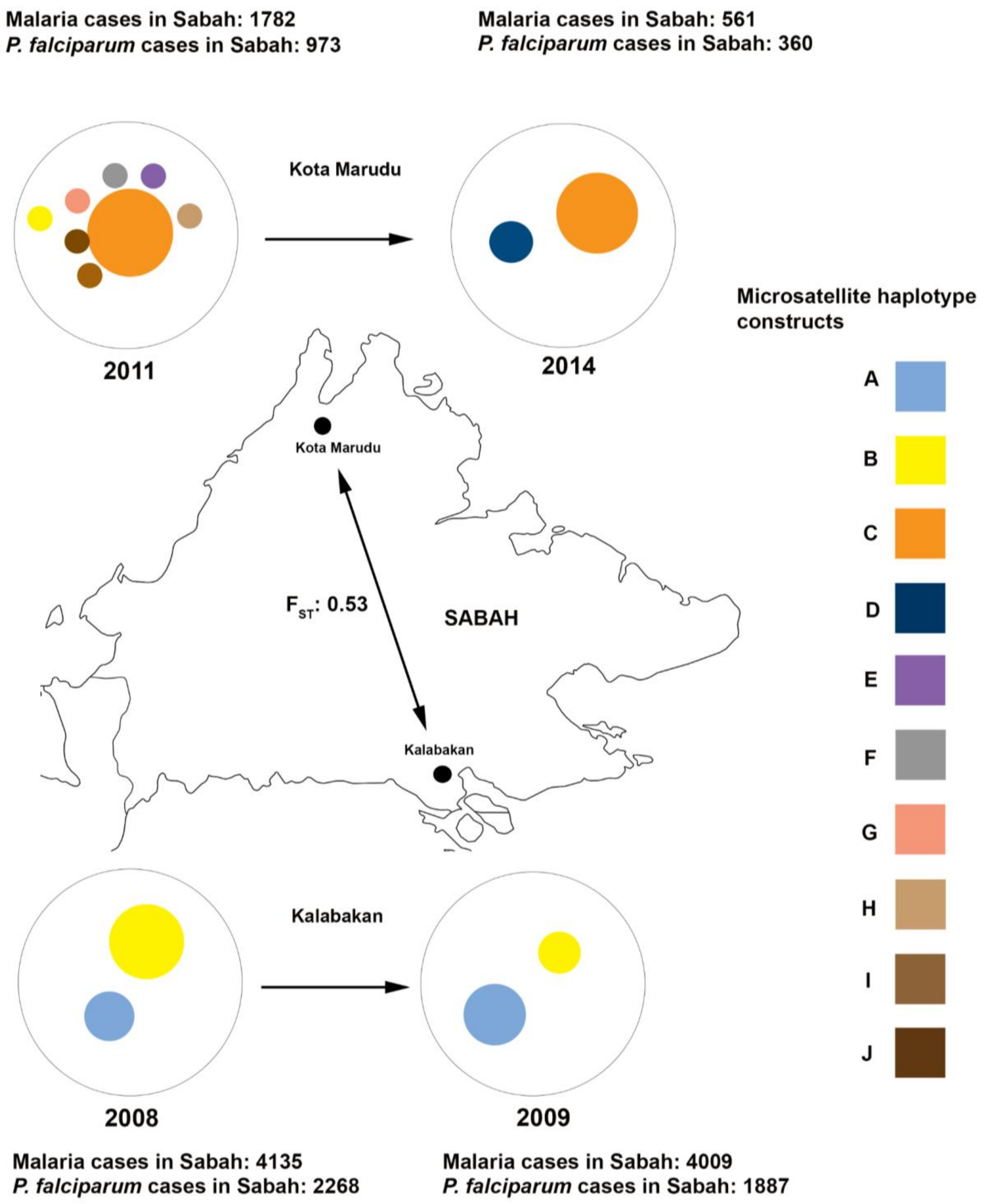

S2 Fig. Divergence between Kalabakan and Kota Marudu *P. falciparum* population structure and the persistence of major genotypes over time based on the distribution of microsatellite haplotype constructs.
