# Supplementary material for: Genetic Diversity of Plasmodium falciparum Populations in Malaria Declining Areas of Sabah, East Malaysia
Source: PLoS One. 2016 Mar 29;11(3):e0152415. doi: 10.1371/journal.pone.0152415 (PMC4811561; doi:10.1371/journal.pone.0152415)
Supplement: S1 Table — (PDF) [file pone.0152415.s003.pdf]

S1 Table. Allele frequencies of each 10 *P. falciparum* microsatellite locus.

Locus	Allele	Kalabakan	Kota Marudu
**PfPK2**	**N**	19	24
	**65**	0.632	0.042
	**71**	0.368	0.792
	**74**	0.000	0.042
	**77**	0.000	0.083
	**80**	0.000	0.042
**TA42**	**N**	18	24
	**186**	0.000	0.042
	**189**	1.000	0.042
	**245**	0.000	0.917
**TA1**	**N**	17	24
	**163**	0.000	0.708
	**166**	1.000	0.250
	**169**	0.000	0.042
**TA81**	**N**	18	24
	**113**	0.333	0.042
	**116**	0.000	0.792
	**119**	0.000	0.042
	**122**	0.000	0.042
	**128**	0.667	0.083
**ARA2**	**N**	19	24
	**65**	0.632	0.042
	**71**	0.368	0.792
	**74**	0.000	0.042
	**77**	0.000	0.083
	**80**	0.000	0.042
**POLYa**	**N**	14	24
	**129**	0.286	0.000
	**145**	0.000	0.042
	**161**	0.000	0.125
	**164**	0.000	0.750
	**176**	0.714	0.083
**PFG377**	**N**	18	24
	**96**	1.000	1.000
**TA87**	**N**	19	24
	**101**	0.684	0.083
	**104**	0.316	0.750
	**107**	0.000	0.083
	**110**	0.000	0.083
**2490**	**N**	18	24
	**82**	0.333	0.917
	**85**	0.667	0.083
**TA60**	**N**	17	24
	**76**	0.000	0.042
	**82**	0.647	0.875
	**85**	0.353	0.042
	**98**	0.000	0.042
