# Supplementary material for: Genetic Diversity of Plasmodium falciparum Populations in Malaria Declining Areas of Sabah, East Malaysia
Source: PLoS One. 2016 Mar 29;11(3):e0152415. doi: 10.1371/journal.pone.0152415 (PMC4811561; doi:10.1371/journal.pone.0152415)
Supplement: S2 Table — (PDF) [file pone.0152415.s004.pdf]

S2 Table. Microsatellite haplotype constructs of Kalabakan and Kota Marudu samples by year.

Sites	PfPK2	TA42	TA1	TA81	ARA2	POLYa	PFG377	TA87	2490	TA60	Haplotype	Year of sampling
Kalabakan	071	189	166	113	071	129	096	104	082	085	A	2009
Kalabakan	065	189	166	128	065	176	096	101	085	082	B	2009
Kalabakan	065	189	166	128	065	176	096	101	085	082	B	2008
Kalabakan	065	189	166	128	065	176	096	101	085	082	B	2008
Kalabakan	065	189	166	128	065	176	096	101	085	082	B	2008
Kalabakan	065	ND	166	128	065	ND	096	101	ND	082		2008
Kalabakan	065	189	166	128	065	176	096	101	085	082	B	2008
Kalabakan	065	189	166	128	065	176	096	101	085	082	B	2008
Kalabakan	065	189	ND	128	065	176	096	101	085	082	B	2008
Kalabakan	065	189	166	128	065	176	096	101	085	082	B	2008
Kalabakan	071	189	166	113	071	ND	096	104	082	085	A	2008
Kalabakan	071	189	166	113	071	129	096	104	082	085	A	2008
Kalabakan	065	189	166	128	065	176	096	101	085	082	B	2008
Kalabakan	071	189	166	113	071	ND	096	104	082	085	A	2008
Kalabakan	065	189	166	128	065	ND	096	101	085	ND		2008
Kalabakan	071	189	166	113	071	129	096	104	082	085	A	2009
Kalabakan	071	189	166	113	071	129	096	104	ND	085	A	2009
Kalabakan	071	ND	ND	ND	071	ND	ND	ND	082	ND		2009
Kalabakan	065	189	166	128	065	176	096	101	085	082	B	2008
Kota Marudu	071	245	163	116	071	164	096	104	082	082	C	2014
Kota Marudu	071	245	163	116	071	164	096	104	082	082	C	2014
Kota Marudu	071	245	163	116	071	164	096	104	082	082	C	2011
Kota Marudu	071	245	163	116	071	164	096	104	082	082	C	2014
Kota Marudu	071	245	163	116	071	164	096	104	082	082	C	2014
Kota Marudu	071	245	163	116	071	164	096	104	082	082	C	2014
Kota Marudu	071	245	163	116	071	164	096	104	082	082	C	2014
Kota Marudu	071	245	166	116	071	164	096	104	082	082	D	2014
Kota Marudu	071	245	166	116	071	164	096	104	082	082	D	2014
Kota Marudu	071	245	163	116	071	164	096	104	082	082	C	2011
Kota Marudu	071	245	163	116	071	164	096	104	082	082	C	2011
Kota Marudu	071	245	163	116	071	164	096	104	082	082	C	2011
Kota Marudu	071	245	163	116	071	164	096	104	082	082	C	2011
Kota Marudu	071	245	163	116	071	164	096	104	082	082	C	2011
Kota Marudu	077	245	163	122	077	145	096	107	082	085	E	2011
Kota Marudu	071	245	163	116	071	164	096	104	082	082	C	2011
Kota Marudu	074	186	166	128	074	176	096	101	085	098	F	2011
Kota Marudu	071	245	166	116	071	161	096	104	082	082	G	2011
Kota Marudu	065	189	166	128	065	176	096	101	085	082	B	2011
Kota Marudu	077	245	163	113	077	161	096	107	082	076	H	2011
Kota Marudu	071	245	166	116	071	164	096	110	082	082	I	2011
Kota Marudu	080	245	169	119	080	161	096	110	082	082	J	2011
Kota Marudu	071	245	163	116	071	164	096	104	082	082	C	2011
Kota Marudu	071	245	163	116	071	164	096	104	082	082	C	2011
